# Facile Cellulose Dissolution and Characterization in the Newly Synthesized 1,3-Diallyl-2-ethylimidazolium Acetate Ionic Liquid

**DOI:** 10.3390/polym9100526

**Published:** 2017-10-18

**Authors:** Hui Zhang, Yaoguang Xu, Yuqi Li, Zexiang Lu, Shilin Cao, Mizi Fan, Liulian Huang, Lihui Chen

**Affiliations:** 1 College of Materials Engineering, Fujian Agriculture and Forestry University, Fuzhou 350002, Fujian, China; huizhang@fafu.edu.cn (H.Z.); ygxu99@163.com (Y.X.); yqli@chemteam.cn (Y.L.); luzexiang@gmail.com (Z.L.); scutcsl@163.com (S.C.); mizi.fan@brunel.ac.uk (M.F.); 2 Nanocellulose and Biocomposites Research Centre, College of Engineering, Design and Physical Sciences, Brunel University London, Middlesex UB8 3PH, UK

**Keywords:** 1,3-diallyl-2-ethylimidazolium acetate, ionic liquid, co-solvent system, solubility of cellulose, electrospinning

## Abstract

A facile cellulose solvent 1,3-diallyl-2-ethylimidazolium acetate ([AAeim][OAc]) with high electrical conductivity has been designed and synthesized for the first time, via a quaternization reaction and ion exchange method. The dissolution characteristics of cellulose in this solvent were studied in detail. Meanwhile, the co-solvent system was designed by adding an aprotic polar solvent dimethyl sulfoxide (DMSO) in [AAeim][OAc]. The effects of temperature and the mass ratio of DMSO to [AAeim][OAc] on the solubility of cellulose were studied. Furthermore, the effects of regeneration on the molecular structure and thermal stability of cellulose were determined by Fourier transform infrared spectroscopy (FT-IR), thermal gravity analysis (TGA) and X-ray diffraction (XRD). The findings revealed that the synthesized ionic liquid (IL) has a relatively low viscosity, high conductivity and a good dissolving capacity for bamboo dissolving pulp cellulose (Degree of Polymerization: DP = 650). The macromolecular chain of the cellulose is less damaged during the dissolution and regeneration process. Due to the increased number of “free” anions [OAc]**^−^** and cations [AAeim]**^+^**, the addition of DMSO can significantly increase the solubility of the cellulose up to 12 wt % at the mass ratio of 3:1, indicating that the synthesized IL has a potential application in the electrospinning field.

## 1. Introduction

During the last decades, ionic liquids (ILs) have received global attention because of their unique properties including high chemical and thermal stability, low vapor pressure and excellent structure designability [1,2,3]. These distinguished advantages have endowed ILs with versatile applications in areas such as biomass transformation [4,5,6], separation and purification [7,8], electrochemistry [9,10], nanomaterials preparation [11,12] and so on.

Besides these potential applications, a series of powerful ILs (e.g., carboxylate-anion and chloride-anion based imidazolium ILs) have been developed to dissolve cellulose [5,13,14]. Meanwhile, Rinaldi indicated that adding an aprotic polar solvent (e.g., dimethyl sulfoxide(DMSO), N,N-Dimethylformamide (DMF) and N-N-Dimethylacetamide (DMAC))to the IL would mean it had lower viscosity and a higher dissolving rate than pure ILs [15]. However, most of the research only focuses on the development of IL solvents with good solubility for cellulose. ILs with the excellent properties of both good solubility and electrical conductivity have been rarely reported. In fact, this type of IL plays a prominent part in the field of electrospinning. Adding ILs into the electrospinning solution will lead to changes in the conductivity, surface tension and viscosity of the solution. It will also determine the feasibility of electrospinning, especially the flow properties of the solution [16,17,18]. In addition, using the conductive ionic liquids as a solvent or co-solvent, the pollution in traditional spinning caused by a volatile organic solvent such as polyacrylonitrile (PAN) or poly (m-phenyleneisophthalamide) (PMIA) can also be reduced accordingly [19]. Therefore, design and synthesis of a type of IL with good solubility, low viscosity and high conductivity for electrospinning is desperately needed.

The physical-chemical properties and some special functions of ILs can be adjusted by designing different structures of anion and cation. Imidazolium-based IL was reported to have high stability and good solubility in cellulose [20,21]. Recent studies have indicated that introducing allyl on the imidazole ring can significantly reduce the melting point and viscosity of ILs, and using the acetic acid anion instead of chlorine anion can improve their solubility [13]. In addition, it was reported that imidazolium-based ILs are not at all inert solvents for cellulose and they react at C-2 with cellulose at its reducing end, forming a carbon–carbon bond [22]. However, by applying 2-alkylsubstituted ILs [23], the reaction with the reducing end of celluloses can be completely avoided. Therefore, we expect to design a new cation structure by introducing two allyl groups and one ethyl group to N-1, N-3 and C-2 of the imidazole ring, respectively. More importantly, the two introduced allyl groups on the imidazole ring can be self-polymerized into ionic liquid conductive polymer materials, which has great potential in electrospinning and polymer lithium batteries, etc.

Based on the above consideration, a facile cellulose solvent, 1,3-diallyl-2-ethylimidazolium acetate ([AAeim][OAc]) with a high electrical conductivity has been firstly designed and synthesized by a quaternization reaction and ion exchange method. The structure and physical properties of [AAeim][OAc] were characterized in detail. The dissolution characteristics of cellulose in the developed solvent were studied in detail. In addition, the [AAeim][OAc]/DMSO co-solvent system has been developed by adding DMSO into the newly synthesized IL [AAeim][OAc] and the dissolving behavior of cellulose in this system was also discussed. Finally, the effects of regeneration on the molecular structure and thermal stability of cellulose were studied by Fourier transform infrared spectroscopy (FT-IR), thermal gravity analysis (TGA) and X-ray diffraction (XRD).

## 2. Materials and Methods

### 2.1. Materials

2-Ethylimidazole (>99%) and Allyl chloride (>98%) were purchased from Aladdin Biochemical Technology Co., Ltd. (Shanghai, China). Tetrahydrofuran (>98%), potassium hydroxide (>98%), silver nitrate (>98%), dimethyl sulfoxide (>98%) and 717-anion-exchang resin were obtained from Sinopharm Chemical Reagent Co., Ltd. (Shanghai, China). Before use, dimethyl sulfoxide was dried by molecular sieve to remove the water. A bamboo dissolving pulp (α-cellulose content of 98.4% and polymerization degree of 650) was kindly supplied by Qingshan Paper Industry Co., Ltd. (Fuzhou, China) and was ball-milled and sieved through a 200-mesh sieve before use.

### 2.2. Synthesis of the IL

**Figure 1:**
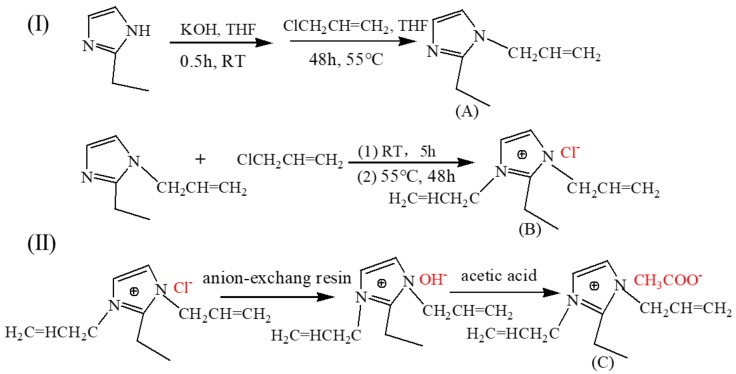
The ionic liquid (IL) of 1,3-diallyl-2-ethylimidazolium acetate was synthesized by a two-step method; step (**I**) Quaternization reaction; step (**II**) Anion exchange process

The solvent of 1,3-diallyl-2-ethylimidazolium acetate ([AAeim][OAc]) was synthesized by a two-step method, as shown in Figure 1I,II. Firstly, the halogen intermediate with two allyls was prepared by a quaternization reaction. Then, the objective product of carboxylate IL was obtained by anion exchange between the acetic acid anion and the halogen ion of the intermediate.

#### 2.2.1. Quaternization Reaction

In brief, 2-ethylimidazole (2.46 g) and potassium hydroxide (0.96 g) were dissolved in 30 mL tetrahydrofuran (THF), to which allyl chloride (the molar ratio of 2-ethylimidazole:allyl chloride = 1:1.2) was added. After 48 h of reaction at 55 °C, the solid impurities of the above system were filtered out. The product of 1-allyl-2-ethylimidazole (A) with the yield of 86% was obtained by removing unreacted allyl chloride via rotary evaporation. Then, a slight excess of allyl chloride was added slowly to the above product with the molar ratio of 1.1:1. After reaction for 5 h at room temperature, the system was heated up to 55 °C and continued to react for 48 h. The product was repeatedly washed with absolute ether to remove the unreacted reactant (A). Finally, the product of 1,3-diallyl-2-ethylimidazolium chloride (B) with the mass yield of 83% was obtained after removal of allyl chloride and ether via rotary evaporation (Figure 1I).

#### 2.2.2. Anion Exchange

In order to obtain [AAeim][OH], the [AAeim][Cl] ethanol solution was pumped into a column filled with 717-anion-exchange resin at a speed of 10 mL/min for 24 h. Meanwhile, at the bottom of the column, the mixture of AgNO_3_-HNO_3_ and PH test paper were used to detect the presence of chloride ions. Before use, 717-anion-exchange resin was activated through alkali–acid–alkali treatment [24]. Then, [AAeim][OH] ethanol solution was neutralized with an equimolar quantity of acetic acid under magnetic stirring in an ice-water bath. To remove residual volatile compounds and water, the obtained [AAeim][OAc] mixtures were purified via rotary evaporation for 72 h (Figure 1II). Finally, the product of [AAeim][OAc] was thoroughly washed with anhydrous ethers, dried under vacuum at 70 °C for 72 h, and stored in a desiccator.

### 2.3. Measurements of Impurity Content of the ILs

The physicochemical properties of the newly synthesized ionic liquid may be severely affected by some impurities, such as free water and Cl^−^, even in trace amounts [25,26]. In addition, free water content significantly affects cellulose solubility. In this study, the free water content in the IL was determined by Karl-Fischer titration (Mettler DL35, Mettler Toledo, Beersel, Belgium) and less than 0.19 ± 0.01 mass-% water (1900 ppm) remained in the IL. The Cl^−^ content in the IL was investigated by a chloride ion-selective electrode (PCL-1, Shanghai Instrument Electric Analytical Instruments Co., Ltd., Shanghai, China) and less than 0.07 ± 0.01 mass-% Chloride (700 ppm) was found in the IL. With reference to the work of Zhao, et al. [26], it was inferred that the free water and Cl^−^ contents that remained in this IL did not noticeably influence the cellulose solubility.

### 2.4. Dissolution of Bamboo Dissolving Pulp Cellulose in the Synthesized [AAeim][OAc] and [AAeim][OAc]/DMSO Co-Solvent System

The co-solvent system of [AAeim][OAc]/DMSO was designed by adding DMSO to dried [AAeim][OAc] with the given mass ratio of IL to DMSO (R_IL/DMSO_ =1:5, 1:3, 1:1, 3:1 and 5:1, respectively). The typical dissolution experiment [27,28,29] was performed (see Supplementary Materials) to measure the solubilities of bamboo dissolving pulp cellulose (DP = 650) in the [AAeim][OAc] and [AAeim][OAc]/DMSO co-solvent system at different temperatures and mass ratios. The dissolution process was assessed by using a Leica DMLP polarizing optical microscope (Leica Company, Wetzlar, Germany). A droplet of the solution was sandwiched between a clean glass slide and a coverslip, in order to observe the dissolving situation. After the dissolution process was finished, the dissolved cellulose in different solvent systems was placed into water for regeneration. Then, the regenerated cellulose from the co-solvent system was characterized by Fourier transform infrared spectroscopy (FT-IR), Thermal gravity analysis (TGA) and X-ray diffraction (XRD).

### 2.5. Characterization

The structure of the developed IL was determined by Fourier Transform infrared spectroscopy (FT-IR) and ^1^H Nuclear Magnetic Resonance testing (^1^H-NMR). In addition, the effects of regeneration on the molecular structure and thermal stability of cellulose were studied by FT-IR, an X-ray diffractometer (XRD) and thermal gravimetric analysis (TGA). A FT-IR spectrometer (Thermo Nicolet 360, Thermo Nicolet Corporation, Madison, WI, USA) with a resolution of 4 cm^−1^ and spectral region of 500–4000 cm^−1^ was used in the test. ^1^H-NMR spectra of the IL were collected from a Bruker Avance-400 NMR spectrometer (Bruker Biospin GmbH, Karlsruhe, Germany) operating at 400 MHz. Before use, the synthesized IL was completely dissolved in the solvent of D_2_O at room temperature. XRD data was collected in a scan mode with the scanning speed of 5°/min in the 2θ range between 5° and 60°. The pattern was Cu-Kα radiation (MiniFlex-2) with a voltage of 40 kV and a current of 30 mA. TGA curves were measured on a TG-DTA instrument (Netzsch STA 449F3, Netzsch Gerätebau GmbH, Selb, Germany). Approximately 5 mg of original or regenerated cellulose was weighed and heated from 30 °C to 550 °C (10 °C/min) with a nitrogen flow rate of 20 mL/min.

### 2.6. Determination of Physical Properties of the Synthesized IL

#### 2.6.1. Solubility Measurement

A certain amount of IL was added to the comparison tubes containing different organic solvents (polar and non-polar), and stirred magnetically for 2 min. After standing for a while, the dissolution performances of IL in different organic solvents were observed.

#### 2.6.2. Viscosity Measurement

Following the works of Song, et al. [30], Rodrigues, et al. [31] and Ren, et al. [32], viscosities of the newly synthesized ILs were measured by a digital rotational viscometer (NDJ-8S, Shanghai Precision & Scientific Instrument Co., Ltd., Shanghai, China) at temperatures ranging from 20 °C to 60 °C with a temperature uncertainty of ±0.1 °C. Each data point of the viscosity is the average value of three measurements. The uncertainty of the viscosity measurement is ±3%.

#### 2.6.3. Conductivity Measurement

The conductivity of the IL with varying water content was measured using a portable conductivity meter at temperatures ranging from 305.15 K to 345.15 K.

## 3. Results and Discussion

### 3.1. Structure of the Synthesized IL

**Figure 2:**
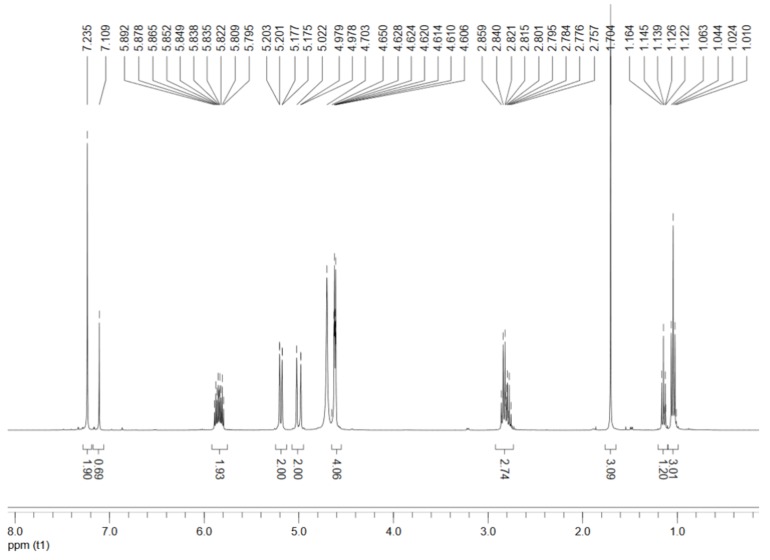
^1^H Nuclear Magnetic Resonance (H-NMR) spectra of the synthesized 1,3-diallyl-2-ethylimidazolium acetate.

The FT-IR spectra of the synthesized 1,3-diallyl-2-ethylimidazolium acetate and 1,3-diallyl-2-ethylimidazolium chloride can be seen in Supplementary Materials Figure S2. Furthermore, the structure of the synthesized 1,3-diallyl-2-ethylimidazolium acetate was confirmed by ^1^H-NMR (Figure 2). The ^1^H-NMR spectra of the IL were collected from a Bruker Avance-400 NMR spectrometer operating at 400 MHz. Before use, the synthesized IL was completely dissolved in the solvent of D_2_O at room temperature. The ^1^H-NMR data of the IL is listed as follows. ^1^H-NMR (400 MHz, D_2_O): δ (ppm) 1.0 (t, 3H, -CH_2_-C**H**_3_) [33], 1.8 (s, 3H, C**H**_3_-COO**^−^**), 2.8 (m, 2H, -C**H**_2_-CH_3_), 4.6 (s, 4H, =CH-C**H**_2_-N**^+^** and N-CH_2_-CH**^−^**), 5.1 (d, 4H, C**H**_2_=CH- and -CH=C**H**_2_), 5.8 (m, 2H, CH_2_=C**H**-CH_2_- and -CH_2_-C**H**=CH_2_), 7.2 (s, 2H, -C**H**=C**H** in imidazole ring).

### 3.2. Physical Properties of the Synthesized IL

**Figure 3:**
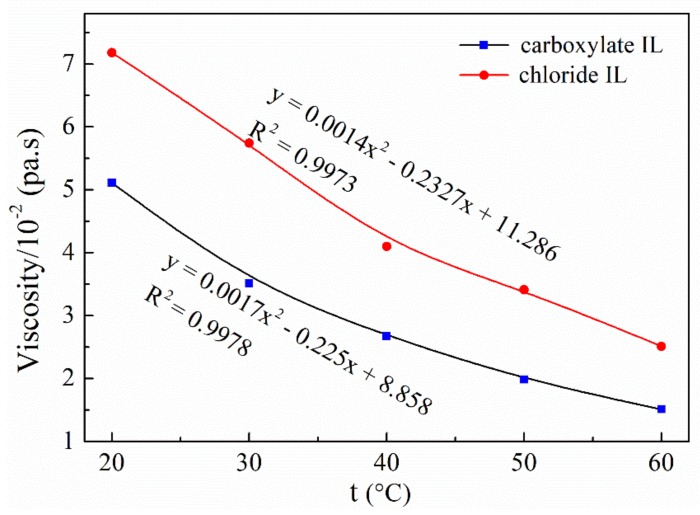
The viscosity of ILs at different temperatures.

The solubilities of IL with some polar and non-polar organic solvents were listed in Supplementary Materials Table S1. Figure 3 shows the viscosities of the developed chloride IL (B) and carboxylate IL (C) at different temperatures.

As seen in Figure 3, both the viscosities of ILs decrease greatly as the test temperature increases in the range from 20 °C to 60 °C. It is evident that the carboxylate IL (C) has a lower viscosity than that of chloride IL (B) at the same temperature. Lower viscosity is beneficial to the diffusion of solvent into the interior of the cellulose molecule. Therefore, the carboxylate IL (C) has a greater solubility for cellulose than the chloride IL (B). To further study the relationship between viscosity and temperature, the mathematical viscosity models for the two ILs were built by fitting the viscosity–temperature data (Figure 3). The R^2^ value associated with the regression was very close to 1, indicating that the predicted values were very close to the actual values of viscosity. Thus, it is very reasonable to predict the viscosities of ILs at different temperatures using the obtained model.

**Figure 4:**
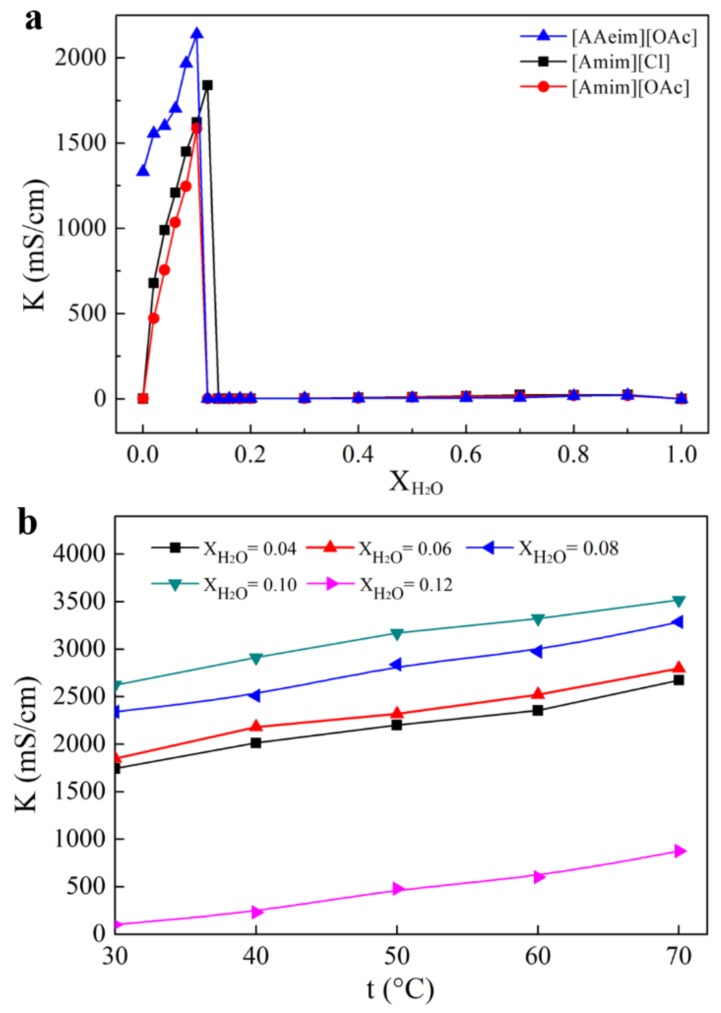
(**a**) The electric conductivities of 1,3-diallyl-2-ethylimidazolium acetate ([AAeim][OAc]), 1-allyl-3-methylimidazolium chloride ([Amim][Cl]) and 1-allyl-3-methylimidazolium acetate ([Amim][OAc]) with different water contents (weight percent of water in the mixture); (**b**) The electric conductivities of 1,3-diallyl-2-ethylimidazolium acetate with different levels of water content (0.04~0.12) and temperature (30~70 °C).

The electrical conductivities of 1,3-diallyl-2-ethylimidazolium acetate, 1-allyl-3-methylimidazolium chloride and 1-allyl-3-methylimidazolium acetate were compared and shown in Figure 4a. Meanwhile, the conductivities of the developed 1,3-allyl-2-ethylimidazolium carboxylate at different temperatures and water contents were also investigated and shown in Figure 4b.

As seen in Figure 4a, the conductivity of pure IL is extremely low, and adding a small amount of water (weight percent < 0.1) in the IL will rapidly enhance its conductivity. This is because the addition of water provides a good ionizing environment for the developed IL, which allows the anion and cation to move freely, resulting in an increase in conductivity. However, continuously increasing water content to a certain extent will lead to a rapid reduction in conductivity, because the dilution effect of water on the IL is dominant. It is obvious that the newly synthesized 1,3-diallyl-2-ethylimidazolium acetate has a higher conductivity than the other two ILs. This is because the newly synthesized diallyl-ethylimidazolium acetate has two allyls, which can be self-polymerized into ionic liquid conductive polymer materials, as we expected in the design. In addition, imidazolium cation in diallyl-ethylimidazolium acetate is a planar structure. Due to this conjugate effect, the charges are uniformly distributed in the whole imidazole ring which weakens the interaction between the cation and the surrounding anion, resulting in a high degree of ion dissociation and a higher conductivity of ionic liquids.

Figure 4b showed the electric conductivities of 1,3-diallyl-2-ethylimidazolium acetate with different levels of water content and temperature. It is quite evident that the conductivities of all the IL-water systems increased as the temperature (from 30 °C to 70 °C) and water content (from 0.04 to 0.10) increased. This could be explained as follows: on the one hand, dissociation is an endothermic process and increasing temperature could improve the ionic dissociation ability of the system and enhance the activity of ions. On the other hand, with the increase of temperature and the molar fraction of the solvent, the viscosity of the IL-water system was greatly reduced, which was favorable for the migration of ions. However, due to the effect of dilution, the electric conductivities began to decrease when the water content increased to 0.12. This result is in good agreement with that obtained in Figure 4a.

### 3.3. Solubilities of Bamboo Dissolving Pulp Cellulose in a [AAeim][OAc] and [AAeim][OAc]//DMSO Co-Solvent System

Polarized optical microscopy was used to probe the dissolution process of bamboo dissolving pulp cellulose in the solvent, as shown in Figure S1. It can be seen that the bamboo dissolving pulp cellulose consisted of many long fibers at the beginning (Figure S1a). As time went on, the number of undissolved fibers decreased (Figure S1b–d). At the end of 30 min, only a small amount of short fibers existed. Finally, no fibers were visible in the field of view. Meanwhile, a relatively homogeneous, clear solution was obtained, indicating that the bamboo dissolving pulp cellulose was fully dissolved in IL solvent.

**Figure 5:**
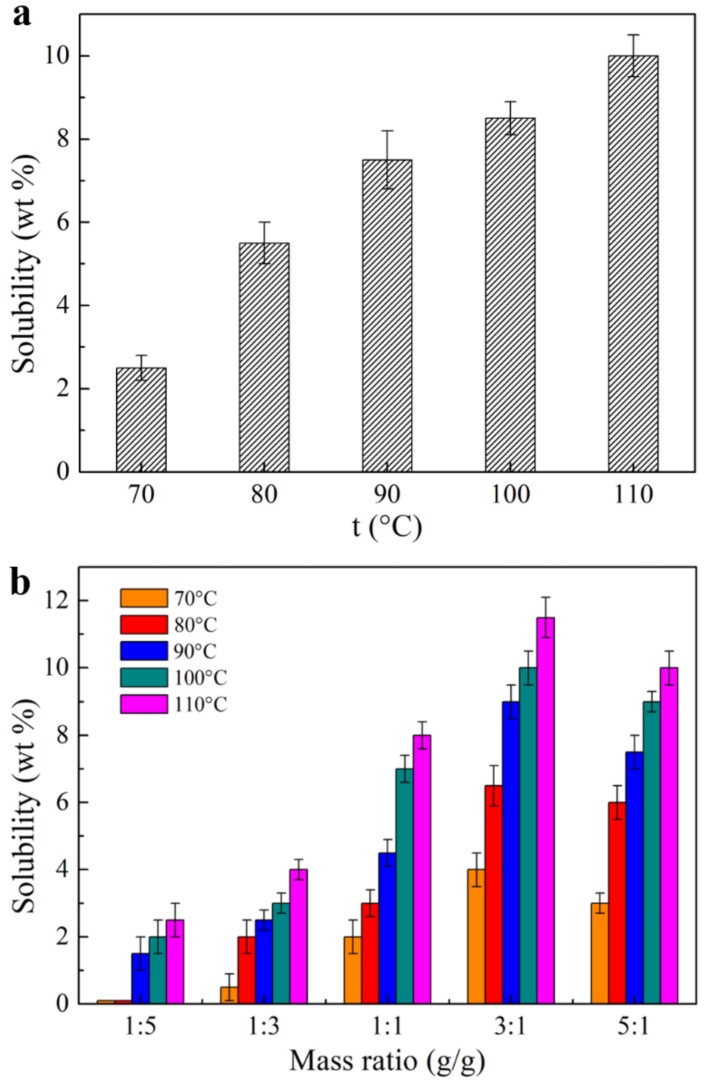
Solubilities (g per 100 g of IL) of bamboo dissolving pulp cellulose (Degree of Polymerization: DP = 650) in (**a**) the synthesized [AAeim][OAc] solvent; and (**b**) [AAeim][OAc]/DMSO (dimethyl sulfoxide)co-solvent system at different temperatures and mass ratios.

Figure 5 shows the dissolution behavior of bamboo dissolving pulp cellulose in the developed [AAeim][OAc] and [AAeim][OAc]/DMSO co-solvent system, respectively. As shown in Figure 5a, the solubility (g per 100 g of IL) of cellulose in the developed IL increased almost five times as the temperature increased from 70 °C to 110 °C. This is because the viscosity of the IL decreases as temperature increases, which is favorable to the swelling and diffusion of cellulose in the [AAeim][OAc] solvent. Moreover, the temperature increment also contributes to breaking the old hydrogen bonds of cellulose, thereby enhancing its solubility. However, the solubility of cellulose in this IL is slightly lower than in the currently reported IL solvent such as [C_4_mim][OAc] and [Amim][OAc]. It may be due to the self-polymerization of the two allyl groups in the developed IL.

In order to further improve the solubility of cellulose, the [AAeim][OAc]/DMSO co-solvent system was designed. Figure 5b shows the solubilities of dissolving pulp cellulose in the [AAeim][OAc]/DMSO co-solvent system at different temperatures and mass ratios. As the temperature increases from 70 °C to 110 °C, the solubility of cellulose in the [AAeim][OAc]/DMSO co-solvent system also greatly improves. It is apparent that the addition of DMSO to [AAeim][OAc] also has a significant effect on solubility. As shown in Figure 5b, the solubility of cellulose increases in the mass ratio ranging from 0 to 3 as DMSO content increases, reaching the maximum solubility in the [AAeim][OAc]/DMSO co-solvent (R_IL/DMSO_ =3:1) at all temperatures studied. The highest solubility is up to 12% at the temperature of 110 °C and mass ratio of 3:1, which is higher than that in the [AmimA][CH_3_COO] solvent without adding DMSO. This can be explained by the addition of DMSO in the developed IL which could partially disassociate the [AAeim][OAc] into an [OAc]^−^ anion and an imidazole cation with allyl, which can readily interact with cellulose and enhance solubility [34,35]. However, the dilution effect on the anion concentration is dominant when the DMSO content increases to a certain extent, resulting in a decrease in solubility.

### 3.4. Structure and Thermal Stability of the Regenerated Cellulose

**Figure 6:**
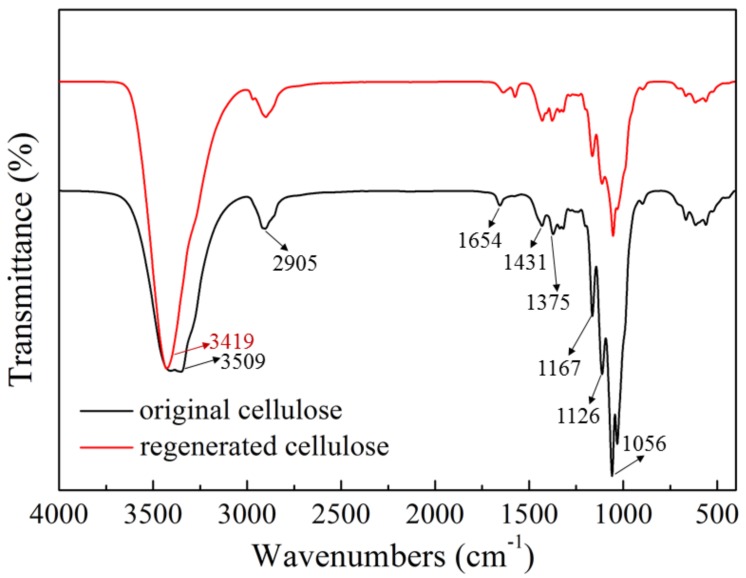
Fourier transform infrared spectroscopy (FT-IR) spectra of the original cellulose, and the cellulose regenerated from the developed [AAeim][OAc]/DMSO co-solvent system.

Figure 6 showed the FT-IR spectra of original and regenerated cellulose. The two spectra were basically the same without new peaks observed in the regenerated cellulose, which demonstrated that the main functional groups in cellulose were not changed and no chemical reaction occurred during the dissolution ([AAeim][OAc]/DMSO co-solvent) and regeneration processes. The peaks located at 3419 cm^−1^ and 2905 cm^−1^ were assigned to the stretching vibration of O–H and C–H band in the cellulose macromolecule, respectively. The bands around 1167 cm^−1^ and 1126 cm^−1^ were caused by stretching vibrations of the C–O–C band in the original cellulose [36]. In addition, the symmetric bending vibration and swing vibration of –CH_2_ could be seen at 1431 cm^−1^ and the bending vibration of the C–H bond could be seen at 1375 cm^−1^. After being regenerated, the O–H vibration shifts to a higher wavenumber (3509 cm^−1^), indicating that the breaking of hydrogen bonds weakened the bonding force during the dissolution [37]. The presence of all of the above bands suggests that the dissolution of cellulose in the [AAeim][OAc]/DMSO co-solvent system is a physical process.

**Figure 7:**
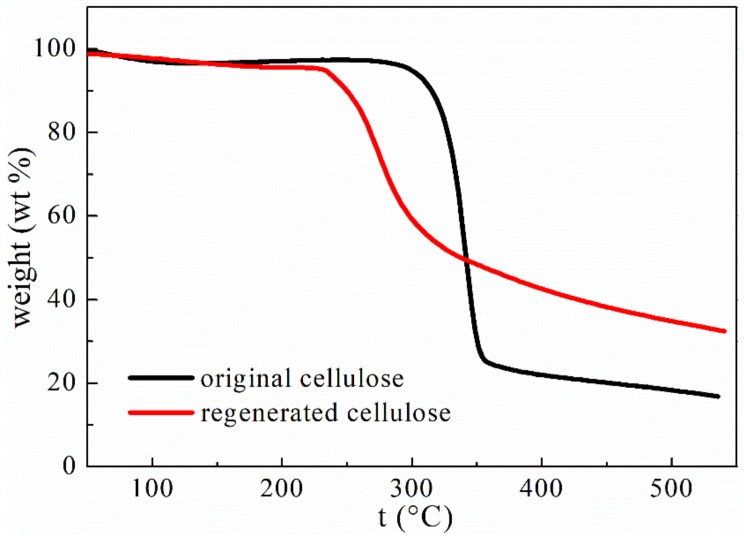
Thermal decomposition profiles of the original cellulose, and the cellulose regenerated from the developed [AAeim][OAc]/DMSO co-solvent system.

Thermal decomposition profiles of the original cellulose and the cellulose regenerated from the developed [AAeim][OAc]/DMSO co-solvent system are shown in Figure 7. It is apparent that the two profiles are almost overlapping at a lower temperature and that the rapid decomposition occurs in the temperature ranging from 285 °C to 351 °C for the original cellulose and 240 °C to 305 °C for the regenerated cellulose. The weight losses over these temperature ranges were mainly caused by the breaking of the glycosidic bonds and part of the glucose units in the cellulose molecules. Compared with the original cellulose (onset temperature T_dcp_ = 285 °C), the regenerated cellulose exhibits a slightly lower onset temperature (T_dcp_ = 240 °C). The lower T_dcp_ means a lower thermal stability. The decrease in the thermal stability of the regenerated cellulose may be attributed to the decreased number of hydrogen bonds between cellulose chains and crystallinity. Similar observations have been reported by Pang et al. [38] and Xu et al. [21] during the dissolution of cellulose in ionic liquid. However, the regenerated cellulose has a slightly higher char yield (nonvolatile carbonaceous material) on pyrolysis than that of the original cellulose, indicated by the slightly higher residual mass after the decomposition step [39].

**Figure 8:**
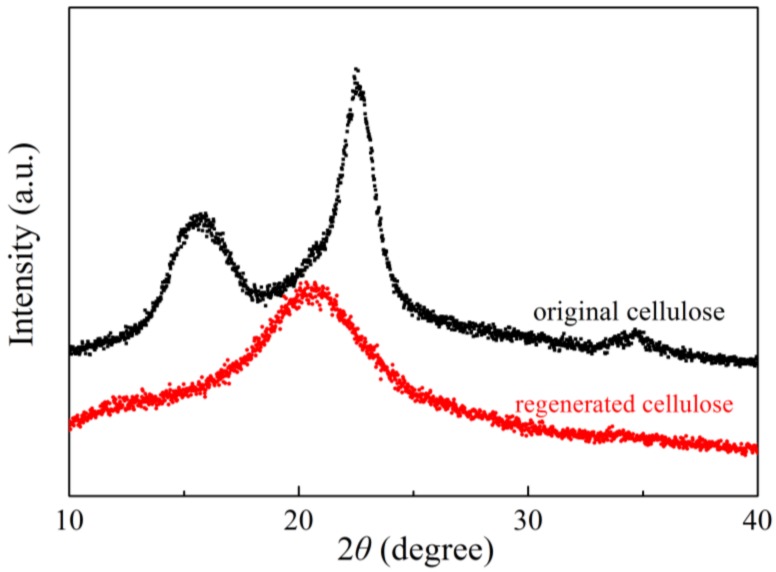
The X-ray diffraction (XRD) spectra of the original cellulose, and the cellulose regenerated from the developed [AAeim][OAc]/DMSO co-solvent system.

Figure 8 shows the XRD spectra of the original and regenerated cellulose. The diffraction peaks at 2θ = 16.50°, 22.96° and 34.84° that appear in the XRD spectra indicate the typical structure of cellulose I [40]. After dissolution and regeneration in the developed [AAeim][OAc]/DMSO co-solvent system, the intensity of the diffraction peaks at 2θ = 13°and 21° was weakened, indicating that the crystallinity of cellulose decreased and its crystalline structure changed from I to II [41]. This may be due to the rapid proton exchange of the hydroxyl groups with the solvent molecules during the cellulose dissolution process, resulting in an unrecovered hydrogen bond network structure. However, the DP of the regenerated cellulose (462) is nearly equal to that of the original bamboo dissolving pulp cellulose (DP = 650), implying that the macromolecular chain of the cellulose is less damaged during the dissolution and regeneration process.

## 4. Conclusions

A facile cellulose solvent of 1,3-diallyl-2-ethylimidazolium acetate ([AAeim][OAc]) was designed and synthesized in this study. As expected, the newly synthesized [AAeim][OAc] has a lower viscosity than [AAeim][Cl], and a higher electric conductivity than common ILs such as 1-allyl-3-methylimidazolium chloride ([Amim][Cl]) and 1-allyl-3-methylimidazolium acetate ([Amim][OAc]). The synthesized [AAeim][OAc] has a good dissolving capacity for cellulose. The maximum solubility for bamboo dissolving pulp (DP = 650) was as high as 10 wt% at 110 °C. The addition of aprotic polar solvent DMSO increased the solubility of the cellulose, and the solubility was up to 12 wt% at the mass ratio of 3:1. The regenerated cellulose has a slightly lower thermal stability, which might be due to the changes of the crystal shape of cellulose. The macromolecular chain of the cellulose was less damaged during the dissolution and regeneration process. The results of this development suggested that the developed [AAeim][OAc] could be a superior candidate for dissolving cellulose in the field of electrospinning.

## Supplementary Materials

The following are available online at http://www.mdpi.com/2073-4360/9/10/526/s1, Figure S1: Polarizing microscope micrographs of the bamboo dissolving pulp cellulose (DP = 650) in the newly synthesized diallyl-ethylimidazolium acetate ([AAeim][OAc]) at 100 °C after (a) 0, (b) 15, (c) 20, (d) 25, (e) 30, and (f) 35 min, Figure S2: The FT-IR spectras of the synthesized 1,3-diallyl-2-ethylimidazolium acetate and 1,3-diallyl-2-ethylimidazolium chloride, Table S1: The solubilities of IL in different organic solvents.
