# Supplementary material for: Facile Cellulose Dissolution and Characterization in the Newly Synthesized 1,3-Diallyl-2-ethylimidazolium Acetate Ionic Liquid
Source: Polymers (Basel). 2017 Oct 18;9(10):526. doi: 10.3390/polym9100526 (PMC6418646; doi:10.3390/polym9100526)
Supplement: Supplementary file 1 [file polymers-09-00526-s001.pdf]

Supplementary Materials: Facile cellulose dissolution and characterization in the new synthetized 1,3-diallyl-2-ethylimidazolium acetate ionic liquid

Hui Zhang [1], Yaoguang Xu [1], Yuqi Li [1], Zexiang Lu [1], Shilin Cao [1], Mizi Fan[1,2], Liulian Huang [1,*] and Lihui Chen [1,*]

[1] College of Materials Engineering, Fujian Agriculture and Forestry University, Fuzhou, Fujian 350002, China; huizhang@fafu.edu.cn (H.Z.); ygxu99@163.com (Y.X.); yqli@chemteam.cn (Y.L.); luzexiang@gmail.com (Z.L.); scutcsl@163.com (S.C); hll65212@163.com (L.H.); fafuclh@163.com (L.C.)

[2] Nanocellulose and Biocomposites Research Centre, College of Engineering, Design and Physical Sciences, Brunel University, UB8 3PH, UK; mizi.fan@brunel.ac.uk(M.F.)

* Correspondence: hll65212@163.com (L.H), fafuclh@163.com (L.C.)

Solubilities of bamboo dissolving pulp cellulose in the synthesized [AAeim][OAc] and [AAeim][OAc]/DMSO co-solvent system

[AmimA][CH3COO]/DMSO co-solvent system was designed by adding DMSO in dried [AAeim][OAc] IL with the given mass ratio of IL to DMSO (1:5, 1:3, 1:1, 3:1 and 5:1 were chosen). In a typical dissolution experiment, dissolving pulp cellulose of 0.5g was added into a 50ml flask with a mechanical stirring, in which IL solvent or IL/DMSO co-solvent system has been added. Then, the flask was immersed in an oil bath and the temperature was controlled to be t ± 0.5 °C. The dissolution process was assessed by using a Leica DMLP polarizing optical microscope (Leica Company, German). Additional cellulose was added until the solution became homogeneous and clear (no fibers were visible in the field of view). The solubilities at various temperatures (70 °C–110 °C) could be calculated from the amount of solvent and the cellulose added.

The micrographs obtained from the polarized microscope were shown in Figure S1.

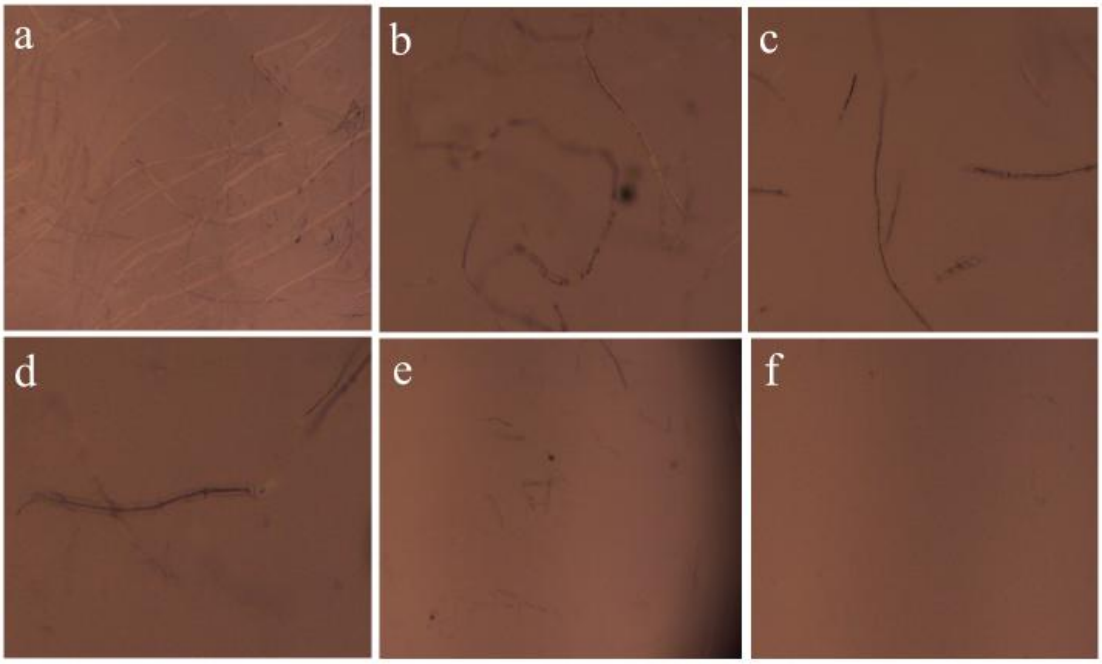

Figure S1. Polarizing microscope micrographs of the bamboo dissolving pulp cellulose (DP = 650) in the newly synthesized diallyl-ethylimidazolium acetate ([AAeim][OAc]) at 100 °C after (a) 0, (b) 15, (c) 20, (d) 25, (e) 30, and (f)35 min.

As can be seen from Figure S1, the bamboo dissolving pulp cellulose consisted of many long fibers at the beginning (Figure S1a). As time went on, the number of undissolved fibers

decreased (Figure S1b-d). At the end of 30min, only a small amount of short fiber existed. Finally, no fibers were visible in the field of view. Meanwhile, a relatively homogeneous clear solution was obtained, indicating that the bamboo dissolving pulp cellulose was fully dissolved in [AAeim][OAc] IL solvent.

Supplementary Figures and Tables

The FT-IR spectras of the synthesized 1,3-diallyl-2-ethylimidazolium acetate and 1,3-diallyl-2-ethylimidazolium chloride were shown in Supplementary Figure. S2.

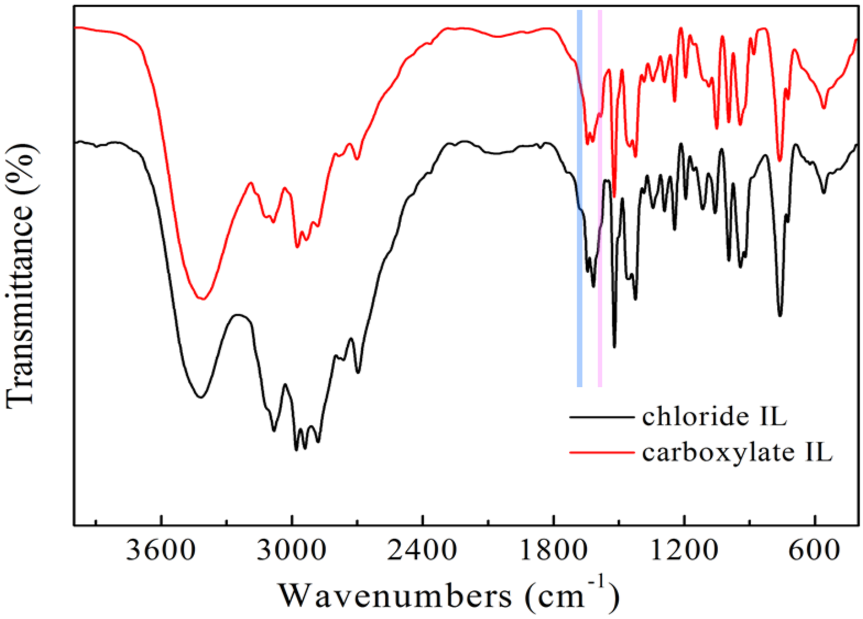

Figure S2 The FT-IR spectras of the synthesized 1,3-diallyl-2-ethylimidazolium acetate and 1, 3-diallyl-2-ethylimidazolium chloride.

By contrast, the two infrared spectra are basically the same except for the difference in the marked region. The peak located from 1643 to1710 cm-1 in carboxylate IL has a greater intensity than that of the chloride IL, which may be caused by skeleton vibration of imidazole ring and the stretching vibration of C=O in –COOH [1-3], indicating that the chloride IL was successfully converted into carboxylate IL. In addition, the C-H out-of-plane bending vibrations of –CH=CH2 could be seen at 999cm-1. The peak located at 1425 cm-1 was assigned to the C-H bending vibration of =CH2. The bands around 1643 cm-1 and 1616 cm-1 were caused by C=C stretching vibration. The bands around 2800 cm-1 and 3082 cm-1 were assigned to the stretching vibrations of C-H band [4]. All the above information confirmed the existence of –CH=CH2 in the synthesized ILs.

The solubilities of IL in different organic solvents were shown in Table S1. It can be found that the developed carboxylate IL is soluble in polar organic solvents (e.g., DMSO, DMAC and chloroform), but insoluble in non-polar solvents (e.g., toluene, ether and ethyl acetate). This might be related to the structure of IL, which make it dissolved in solvents of similar polarities.

Table S1 The solubilities of IL in different organic solvents

**Solvent**	**H_2O**	**DMSO**	**DMF**	**DMAC**	**methanol**	**ethanol**	**chloroform**
Solubility	Y	Y	Y	Y	Y	Y	Y

**Solvent**	**dichloromethane**	**acetonitrile**	**ethyl acetat**	**toluene**	**ether**	**cyclohexane**	**benzene**
Solubility	Y	-	N	N	N	N	N

Note: Y: soluble, homogeneous and clear solution; N: insoluble, layered; -: insoluble, emulsion

References

1. Zhang, F.; Zhang, W.; Yu, Y.; Deng, B.; Li, J.; Jin, J. Sol-gel preparation of PAA-g-PVDF/TiO2 nanocomposite hollow fiber membranes with extremely high water flux and improved antifouling property. *J. Membrane Sci.* **2013**, *432*, 25–32.
2. Madaeni, S. S.; Zinadini, S.; Vatanpour, V. A new approach to improve antifouling property of PVDF membrane using in situ polymerization of PAA functionalized TiO2 nanoparticles. *J. Membrane Sci.* **2011**, *380*, 155–162.
3. Wu, Y.; Liu, X.; Meng, M.; Lv, P.; Yan, M.; Wei, X.; Li, H.; Yan, Y.; Li, C. Bio-inspired adhesion: Fabrication of molecularly imprinted nanocomposite membranes by developing a hybrid organic-inorganic nanoparticles composite structure. *J. Membrane Sci.* **2015**, *490*, 169–178.
4. Lassègues, J.; Grondin, J.; Cavagnat, D.; Johansson, P. New Interpretation of the CH Stretching Vibrations in Imidazolium-Based Ionic Liquids. *J. Phys. Chem. A* **2009**, *113*, 6419–6421.
